# Plasma membrane restricted RhoGEF activity is sufficient for RhoA-mediated actin polymerization

**DOI:** 10.1038/srep14693

**Published:** 2015-10-05

**Authors:** Jakobus van Unen, Nathalie R. Reinhard, Taofei Yin, Yi I. Wu, Marten Postma, Theodorus W.J. Gadella, Joachim Goedhart

**Affiliations:** 1 Swammerdam Institute for Life Sciences, Section of Molecular Cytology, van Leeuwenhoek Centre for Advanced Microscopy, University of Amsterdam, P.O. Box 94215, NL-1090 GE Amsterdam, The Netherlands; 2 Center for Cell Analysis and Modeling, University of Connecticut Health Center, 400 Farmington Avenue, Farmington, CT 06032-6406.

## Abstract

The small GTPase RhoA is involved in cell morphology and migration. RhoA activity is tightly regulated in time and space and depends on guanine exchange factors (GEFs). However, the kinetics and subcellular localization of GEF activity towards RhoA are poorly defined. To study the mechanism underlying the spatiotemporal control of RhoA activity by GEFs, we performed single cell imaging with an improved FRET sensor reporting on the nucleotide loading state of RhoA. By employing the FRET sensor we show that a plasma membrane located RhoGEF, p63RhoGEF, can rapidly activate RhoA through endogenous GPCRs and that localized RhoA activity at the cell periphery correlates with actin polymerization. Moreover, synthetic recruitment of the catalytic domain derived from p63RhoGEF to the plasma membrane, but not to the Golgi apparatus, is sufficient to activate RhoA. The synthetic system enables local activation of endogenous RhoA and effectively induces actin polymerization and changes in cellular morphology. Together, our data demonstrate that GEF activity at the plasma membrane is sufficient for actin polymerization via local RhoA signaling.

Rho GTPases belong to the Ras superfamily of small G proteins and are involved in a variety of cellular processes, such as the dynamic regulation of the actin cytoskeleton and cell morphology, cell cycle progression, and gene transcription^1^,^2^. It is well known that dysregulation of Rho GTPase function plays a key role in tumor formation, invasion and metastasis^3^,^4^. Accumulating evidence points towards Rho GTPases and their effectors and regulators as possible therapeutic targets. Better understanding of the spatiotemporal regulation of Rho GTPase signaling could increase therapeutic success and help in the design of novel therapeutic intervention strategies^5^,^6^.

Like most typical G proteins, Rho GTPases function as molecular switches by cycling between an inactive GDP-bound state and an active GTP-bound state^7^. Three classes of accessory proteins that control the molecular switch kinetics and the location of Rho GTPases in cells have been identified^8^,^9^. Rho guanine exchange factors (Rho GEFs) stimulate the exchange of GDP for GTP, resulting in Rho GTPase activation. In contrast, Rho GTPase-activating proteins (Rho GAPs) accelerate the hydrolysis of bound GTP to GDP, which abrogates Rho GTPase signaling. Inactive, GDP-bound Rho GTPases are sequestered in the cytoplasm by Rho guanine nucleotide dissociation inhibitors (Rho GDIs). The signaling output of Rho GTPases is dictated by spatiotemporal control of GEF and GAP activity and the subcellular location of the Rho GTPase itself.

There are 22 Rho GTPases identified in humans, of which RhoA, Rac1 and Cdc42 have been studied in most detail^10^. RhoA has been linked to the regulation of cytoskeletal dynamics, cell migration and cell adhesion^2^. RhoA is localized to the cytosol in mammalian cells and has been reported to translocate to the plasma membrane upon activation^11^. However, the precise subcellular site and kinetics of RhoA activation by its GEFs is still under investigation.

P63RhoGEF (encoded by the gene ARHGEF25) is a RhoA specific guanine exchange factor^12^,^13^, member of the Dbl superfamily of Rho GEFs. Members of this superfamily are characterized by one or more Dbl-homology (DH) domains, which are almost always accompanied by a C-terminal Pleckstrin Homology (PH) domain^14^. The DH domain interacts directly with the Rho GTPase and is responsible for the catalytic activity that accelerates the exchange of GDP for GTP on the Rho GTPase^7^. Indeed, the catalytic DH domain of p63RhoGEF was shown to be necessary and sufficient for its downstream signaling function^15^, as is the case for many other GEFs.

The role of the PH domain is less clearly defined. It has been hypothesized to assist in plasma membrane localization, facilitate Rho GTPase activation, mediate target specificity, function as scaffold for signaling proteins and/or phospholipids, or autoinhibit the catalytic DH-domain^7^. Interestingly, the PH domain of p63RhoGEF has been shown to exhibit an inhibitory function by preventing the DH domain from accessing RhoA^16^,^17^. By using biochemical, structural and *in vitro* approaches it has been shown that activation of the heterotrimeric G-protein Gαq allosterically activates the GEF activity of p63RhoGEF by binding to the PH domain, which structurally relieves the DH domain from its auto-inhibited state^16^,^18^.

Based on the fact that plasma membrane localization of p63RhoGEF is important for its effective interaction with Gαq^19^,^20^, we set out to investigate the requirement of plasma membrane localization of p63RhoGEF for the activation of RhoA and subsequent downstream signaling. We have used live cell fluorescent imaging techniques and a novel optimized high-contrast FRET-based RhoA biosensor to determine the kinetic parameters of RhoA activation by p63RhoGEF via stimulation of endogenous Gαq-mediated GPCRs in single living cells. Furthermore, we have employed a rapamycin-dependent heterodimerization system to create a plasma membrane recruitable RhoGEF, enabling direct spatiotemporal control over the subcellular localization of Rho GEF activity in mammalian cells. This system is combined with several read-outs based on RhoA activity, actin polymerization and cellular morphology to arrive at the conclusion that RhoGEF activity at the plasma membrane is sufficient for the activation of RhoA, resulting in actin polymerization.

## Results

### An improved FRET based biosensor for RhoA reveals rapid activation of p63RhoGEF after stimulation of an endogenous Gαq-coupled GPCR

**Figure 1:**
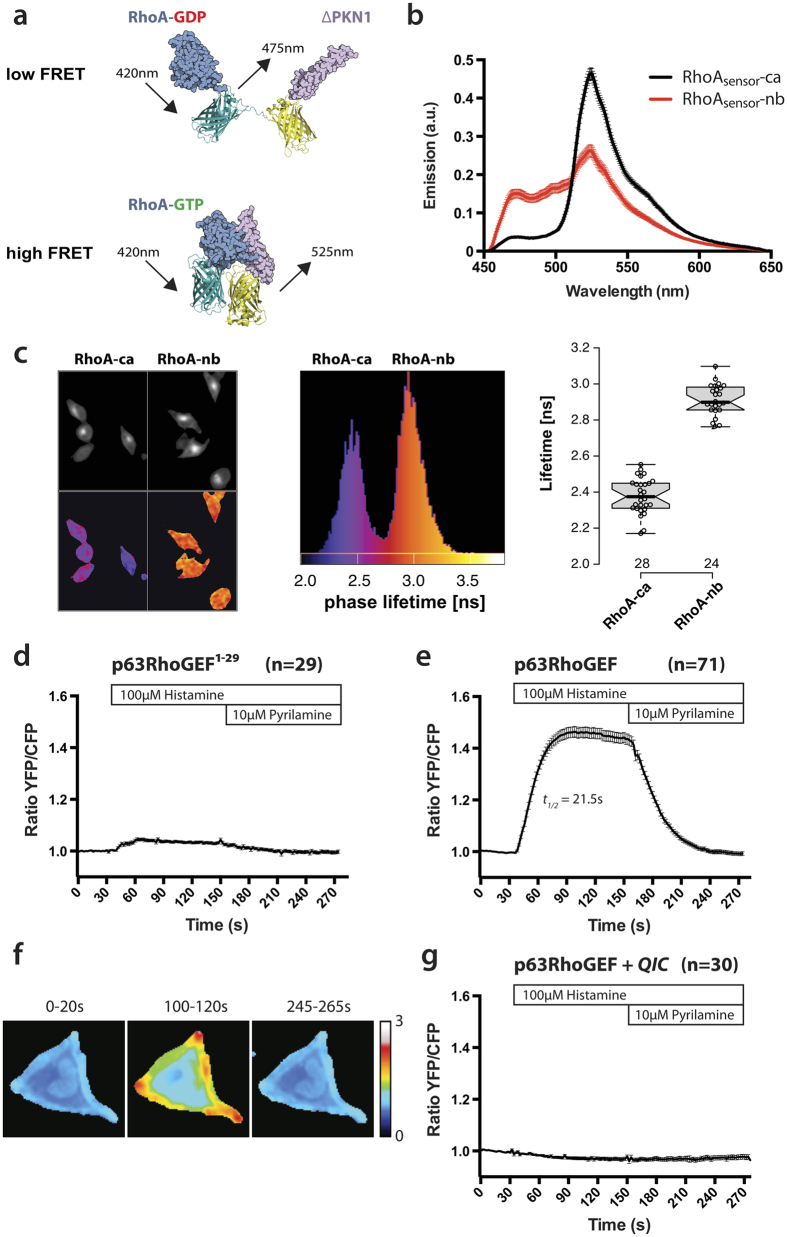
Rapid and reversible GPCR mediated GTP loading of RhoA by p63RhoGEF, measured by a novel high contrast RhoA FRET biosensor. (**a**) A cartoon of the DORA-RhoA biosensor consisting of full length RhoA (shown in *light blue*) fused to CFP, connected via a linker to YFP fused to the Rho binding domain of PKN1 (shown in *lila*) (structures are based on pdb entries 1CXZ, 1MYW and 3ZTF). (**b**) Average emission spectra (±s.e.m) acquired from living cells (n = 10) of the non-binding RhoA biosensor (RhoA_sensor_-nb) and the mutant constitutive GTP loaded RhoA biosensor (RhoA_sensor_-ca). (**c**) (*left*) Donor intensity images (top) and phase lifetime images (bottom) of the RhoA_sensor_-ca (left) and the RhoA_sensor_-nb (right) with a false-color coded lifetime according to the scale depicted in the combined lifetime histograms of the same experiment (*middle*). (*right*) Accumulated FLIM data for RhoA_sensor_-ca and RhoA_sensor_-nb, showing the median phase lifetime from multiple cells (at least 8 acquisitions, n = 28 and n = 24, respectively). Box limits indicate the 25th and 75th percentiles as determined by R software; whiskers extend 1.5 times the interquartile range from the 25th and 75th percentiles. (**d**) Control FRET ratio-imaging experiments in HeLa cells transfected with DORA-RhoA biosensor and only the first 29 a.a. of p63RhoGEF, containing the plasma membrane targeting sequence, show minimal changes in YFP/CFP ratio (n = 29). (**e**) Time-lapse FRET ratio imaging of HeLa cells transfected with the DORA-RhoA biosensor and RFP-p63RhoGEF (n = 71) show fast reversible increase in YFP/CFP ratio, indicating rapid GTP loading of RhoA upon GPCR stimulation. (**f**) Average ratio images at three time intervals of a single cell from the experiment shown in (**e**). (**g**) Pre-incubation with the Gαq-inhibitor QIC (2 μM) abolishes the DORA-RhoA biosensor response by GPCR stimulation in RFP-p63RhoGEF transfected cells (n = 30). HeLa cells were stimulated with Histamine (100 μM) at t = 40 s and the response was antagonized by the addition of Pyrilamine (10 μM) at t = 160 s. Time traces show the average ratio change of YFP/CFP fluorescence (±s.e.m). Average curves consist of data from at least 3 independent experiments, conducted on different days. Width of the individual images in (**f**) corresponds to 65 μm.

The kinetics of the guanine exchange reaction of p63RhoGEF on RhoGTPases have so far only been characterized *in vitro*, using purified proteins^16^. To investigate the spatial and temporal aspects of RhoA nucleotide binding state in individual living cells, a Dimerization Optimized Reporter for Activation (DORA) RhoA biosensor was employed. The single chain FRET based sensor is based on a previously reported RhoA biosensor^21^. When RhoA-GDP is converted to RhoA-GTP a PKN1 moiety binds RhoA-GTP, resulting in a high FRET state, which is detected as an increase in sensitized emission over CFP ratio (Fig. 1a). To examine the FRET contrast between the ‘on’ and ‘off’ state of the biosensor, the emission spectra of a non-binding biosensor (RhoA_sensor_-nb), containing a mutation in PKN1 (L59Q) preventing RhoA binding, and a biosensor containing a constitutively GTP-loaded RhoA mutant (Q63L) (RhoA_sensor_-ca) were measured in HeLa cells. The average single cell spectra show clearly the CFP emission around 475 nm and the (sensitized) YFP emission around 530 nm (Fig. 1b). The RhoA_sensor_-ca shows a marked decrease in CFP emission and an increase in YFP emission relative to the RhoA_sensor_-nb, demonstrating substantial FRET contrast that allows differentiation between the two states. FLIM measurements of the RhoA_sensor_-ca and RhoA_sensor_-nb showed that the donor fluorescence lifetime of the constitutively active mutant is reduced compared to that of the non-binding version (median values change from 2.9 to 2.4 ns), demonstrating that the RhoA-GTP state is accompanied by an increase in FRET efficiency (Fig. 1c).

To examine whether p63RhoGEF is capable of activating RhoA in living cells, we performed live cell measurements of HeLa cells transfected with the DORA-RhoA biosensor and co-transfected with either full length p63RhoGEF or a control construct containing only the first 29 amino acids of p63RhoGEF (p63RhoGEF1–29) (Supplemental Fig. S1). p63RhoGEF1–29 contains only the plasma membrane targeting sequence of p63RhoGEF, and is catalytically inactive. Activation of p63RhoGEF by endogenously expressed histamine-1 receptors (H1R) was achieved by stimulating cells with histamine at the indicated time points, and the response was antagonized by addition of the H1R specific antagonist mepyramine.

Control experiments on cells co-transfected with p63RhoGEF1–29 showed only a minimal change in FRET ratio upon addition of histamine and mepyramine (Fig. 1d). This small observed response could be attributed to the GTP-loading of RhoA by endogenously expressed, Gαq mediated GEFs, like Trio^22^.

A rapid increase in YFP/CFP ratio (30–60%) was observed in the cells transfected with p63RhoGEF with *t*_*½*_ = 21.5 ± 0.7 s after stimulation with histamine, reflecting an increase in RhoA-GTP levels (Fig. 1e). After addition of mepyramine, the response decreased and the YFP/CFP ratio returned to baseline levels. Single cell ratio images from different time points are shown in Fig. 1f. These images suggest that the RhoA biosensor is predominantly activated at the cell periphery.

To verify that the observed effects were mediated by Gαq and not by other G proteins, we used the Gαq specific inhibitor UBO-QIC, a small molecule inhibitor similar to YM-254890^23^,^24^. In cells transfected with p63RhoGEF and pre-incubated with 2 μM UBO-QIC, no RhoA biosensor response to either histamine or mepyramine was observed, confirming that Gαq mediates the response to histamine (Fig. 1g).

During the course of our study, Pertz and co-workers published an optimized RhoA sensor based on teal fluorescent protein (TFP) and Venus^25^, referred to as RhoA2G. A comparison in cells revealed that the DORA-RhoA sensor shows increased brightness relative to RhoA2G and a better average dynamic range in relevant live cell imaging experiments (for details see Supplemental Note).

Thus, p63RhoGEF shows rapid and reversible GEF activity towards an optimized high-contrast RhoA FRET based sensor upon stimulation of H1R mediated Gαq signaling in HeLa cells.

### GEF activity of p63RhoGEF at the plasma membrane is sufficient to induce actin polymerization

P63RhoGEF is located at the plasma membrane by palmitoylation of its cysteine residues near the N-terminus^19^,^20^. To examine whether the plasma membrane location is important for its function, we investigated the effect of multiple p63RhoGEF deletion constructs on actin polymerization.

**Figure 2:**
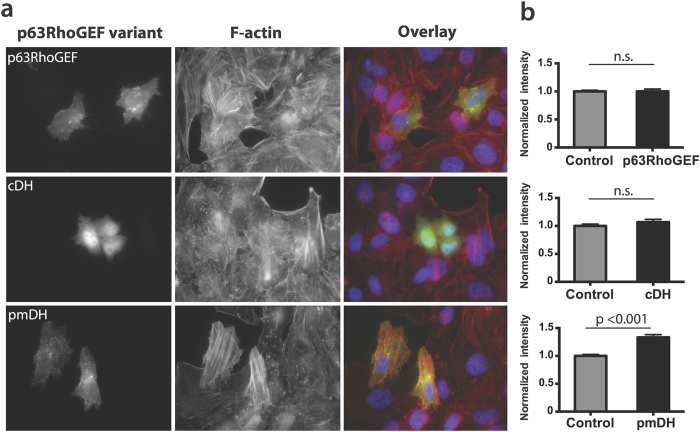
GEF activity at the plasma membrane increases actin polymerization. (**a**) HeLa cells transiently transfected with YFP-p63RhoGEF, YFP-cDH or YFP-pmDH constructs were stained for F-actin after 24 hours with TRITC-phalloidin and DAPI. The panels show from left to right YFP fluorescence, indicating the transfected cells, F-actin staining and the overlay of YFP, actin and DAPI (**b**) Quantification of F-actin in HeLa cells by determining the fluorescent intensity of the TRITC-phalloidin staining in transfected cells and normalization to the intensity of untransfected control cells in the same experiment. YFP-p63RhoGEF n = 18 (control n = 143), YFP-cDH n = 11 (control n = 46), YFP-pmDH n = 32 (control n = 134). Statistical significance per condition was determined by performing a two-tailed student T-test. Width of the individual images in (**a**) is 236 μm.

Since it is well documented that removal of the autoinhibitory PH domain results in increased GEF activity^16^,^18^,^19^, we generated two truncated variants of p63RhoGEF, indicated as plasma membrane DH domain (pmDH) and cytosolic DH domain (cDH), both lacking the PH domain (for details see Supplemental Fig. S1). The cDH construct also does not contain the N-terminal plasma membrane targeting sequence and resides in the cytoplasm, while the pmDH construct is localized like full-length p63RhoGEF, at the plasma membrane. We transfected HeLa cells with either full-length p63RhoGEF or one of its truncated variants. After 24 hours the cells were fixed and stained with an F-actin marker in order to analyze the effect of the different p63RhoGEF constructs on actin polymerization. We noticed that pmDH expression strongly induced actin polymerization, whereas only minimal effects on actin were observed in the full-length p63RhoGEF or cDH conditions (Fig. 2a). This effect was analyzed quantitatively by comparing the fluorescence intensities of the phalloidin staining between the different conditions, which showed a significant difference in fluorescence intensity between transfected and control cells in the pmDH condition (p < 0.001) (Fig. 2b). From this we can deduce that p63RhoGEF guanine exchange factor activity at the plasma membrane results in actin polymerization, while p63RhoGEF guanine exchange factor activity in the cytoplasm does not.

### GEF activity of p63RhoGEF towards RhoA is enhanced at the plasma membrane

Actin polymerization and the often used SRF-activity assays^26^ are a relatively indirect measurement for the activation of RhoA, which might be under influence of considerable crosstalk and signal amplification or desensitization. Therefore, we compared the response of full-length p63RhoGEF and the cDH and pmDH truncated variants on the DORA-RhoA biosensor to evaluate the influence of subcellular localization on GEF activity towards RhoA in a more direct manner.

We transfected HeLa cells with the DORA-RhoA-biosensor and either one of the p63RhoGEF constructs or a control vector that expressed only a red fluorescent protein (RFP), and measured the YFP/CFP FRET ratio to assess the basal state of RhoA activation in each condition. The minimal and maximal ratios were estimated from the inactive RhoA_sensor_-nb probe and the active RhoA_sensor_-ca probe, respectively. The experimental values of the wild-type RhoA-biosensor are expected to fall within this range of YFP/CFP ratios.

**Figure 3:**
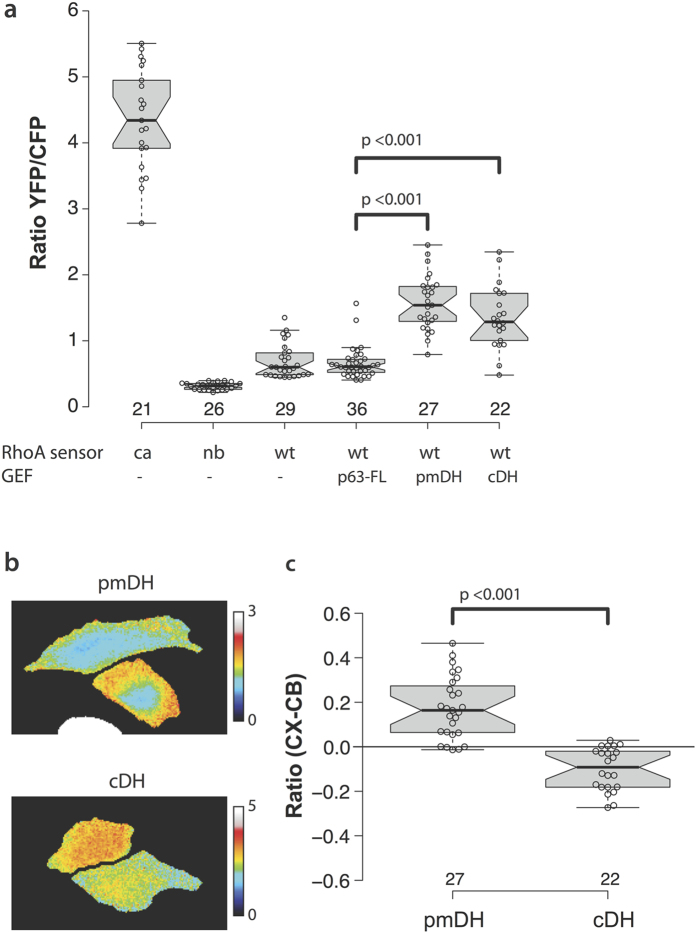
Expression of the differentially localized DH domains of p63RhoGEF, pmDH and cDH, increases Rho-GTP levels with opposite spatial distributions. (**a**) Boxplot showing the basal YFP/CFP ratio of the DORA RhoA biosensor in HeLa cells. Cells transfected with the constitutive active (ca, *n* = 21) or non-binding (nb, *n* = 26) RhoA biosensor were co-transfected with an empty vector containing just RFP to keep expression levels equal between the different experimental conditions. Wild-type (wt) RhoA biosensor was transfected with an empty vector containing just RFP (control, *n* = 29), RFP-p63RhoGEF (*n* = 36), RFP-pmDH (*n* = 27) or RFP-cDH (*n* = 22). (**b**) Representative ratio images of the pmDH (*top panel*) and cDH (*bottom panel*) conditions from the experiment depicted in (**a**), showing the gradient of RhoA GTP loading state in HeLa cells. (**c**) Quantification of the spatial distribution of RhoA GTP loading state between the cell cortex (CX) and the cell body (CB) in the pmDH and cDH conditions of the experiment shown in panel (**a**). The difference value “ratio (CX-CB)” plotted on the y-axis is a measure of spatial inhomogeneity. A value of zero indicates no spatial differences, while a positive value indicates increased RhoA-GTP in the cortex relative to the cell body and a negative value denotes decreased RhoA-GTP in the cortex relative to the cell body (for detailed methods see Supplemental Fig. S3). For boxplots in (**a**,**c**); center lines represent the median values; box limits indicate the 25th and 75th percentiles as determined by R software; whiskers extend 1.5 times the interquartile range from the 25th and 75th percentiles; data points are plotted as open circles. Statistical significance between conditions was determined by performing a two-tailed Mann-Whitney test. P-values are shown in plot for the RhoA-wt biosensor conditions with significant different median values. Width of the individual images in (**b**) is 120 μm.

The YFP/CFP ratios of the RhoA-biosensor in the condition with wild-type p63RhoGEF did not differ significantly from the control condition with cells containing a vector expressing only an RFP (Fig. 3a). In contrast, we found that cells expressing pmDH and cDH had significantly higher YFP/CFP ratios (p < 0.001) on average than cells expressing full-length p63RhoGEF (Fig. 3a). We repeated this experiment in HEK293 cells, which confirmed that cells expressing the pmDH or cDH have a significant higher YFP/CFP ratio than cells expressing p63RhoGEF (Supplemental Fig. S2). This is in agreement with earlier reports of the auto-inhibitory function of the PH domain as tested *in vitro* using purified proteins^16^.

The finding that the cDH and pmDH expressing cells show similar FRET ratios is surprising, since only cells transfected with pmDH showed increased actin polymerization (Fig. 2a,b). Therefore, we decided to examine the location of RhoA GTP-loading activity. Careful examination revealed different gradients of RhoA activity between cells in the pmDH and cDH condition. HeLa cells expressing the pmDH construct show enriched activity around the cell cortex (Fig. 3b), while cells expressing the cDH domain showed an inverted spatial distribution with higher activity in the cell body (Fig. 3b). In order to quantify this effect we subtracted the FRET ratio of a 5-pixel wide region corresponding to the cell cortex from the FRET ratio in the cell body (see Supplemental Fig. S3 for methodological details). We found a significant difference (p < 0.001) in the spatial distribution of RhoA activity between the pmDH and cDH conditions (Fig. 3c).

These results provide evidence for the importance of the plasma membrane localization of RhoGEF activity for its signaling efficiency towards its downstream effectors. The difference between the cDH and pmDH condition in the actin polymerization assay in connection with the spatial difference in the DORA-RhoA biosensor read-out provides evidence for the plasma membrane as platform for RhoA mediated actin polymerization.

### Recruitment of the DH domain of p63RhoGEF to the plasma membrane is sufficient for sustained activation of RhoA

To enable the direct comparison between the effects conferred by cDH and pmDH, we used a rapamycin-dependent recruitment strategy^27^. This strategy allows us to first measure the effects of cytosolic GEF activity and subsequently the effects of plasma membrane located GEF activity, within the same cell.

**Figure 4:**
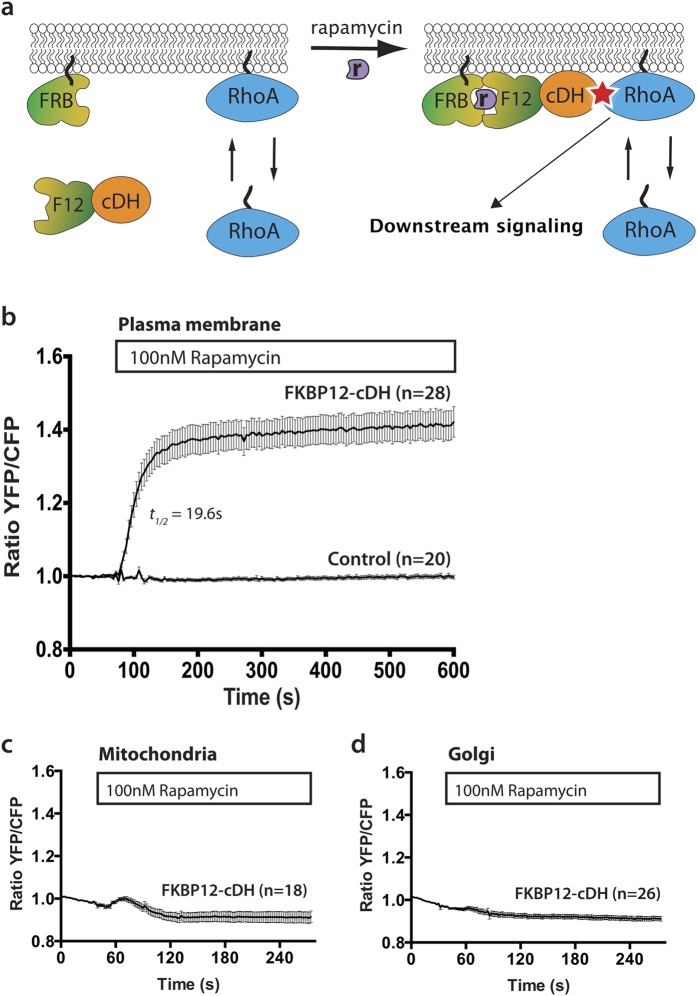
Recruitment of the DH-domain of p63RhoGEF to the plasma membrane is sufficient for sustained activation of RhoA. (**a**) Cartoon illustrating the principle of the recruitment experiment. Rapamycin recruits the cDH domain fused to FKBP12 (F12) to the FRB domain anchored at a membrane, thereby enhancing the local activation. (**b**) Hela cells transfected with the DORA-RhoA biosensor, Lck-FRB-CFP (W66A) (plasma membrane) and RFP-FKBP12-cDH (*n* = 28) or RFP-FKBP12 (control, *n* = 20) were stimulated with Rapamycin (100 nM) at *t* = 60 s. (**c**) Hela cells transfected with the DORA-RhoA biosensor, MoA-FRB-CFP (W66A) (mitochondria) and RFP-FKBP12-cDH (*n* = 18) were stimulated with Rapamycin (100 nM) at *t* = 40 s. (**d**) Hela cells transfected with the DORA-RhoA biosensor Giantin-FRB-CFP (W66A) (golgi apparatus) and RFP-FKBP12-cDH (*n* = 26) were stimulated with Rapamycin (100 nM) at *t* = 40 s. Time traces show the average ratio change of YFP/CFP fluorescence (±s.e.m). Average curves consist of data from at least 3 independent experiments, conducted on different days.

RFP-cDH or RFP constructs were fused to an FKBP12 domain, which, upon addition of rapamycin, will be recruited to Lck-FRB-ECFP (W66A). Lck-FRB-ECFP (W66A) is a plasma membrane localized FRB construct fused to a non-fluorescent ECFP variant (Fig. 4a).

We transfected HeLa cells with the DORA-RhoA biosensor, Lck-FRB-ECFP (W66A) and either RFP-FKBP12-cDH or RFP-FKBP12. Rapamycin induced recruitment of RFP-FKBP12-cDH to the plasma membrane resulted in a rapid and sustained increase in GTP loaded RhoA, as measured by the increasing YFP/CFP ratio of the RhoA biosensor over time (Fig. 4b). The response on the RhoA biosensor was fast with *t*_*½*_ = 19.6 ± 0.4 s and resulted in an average 40% ratio change compared to the baseline, comparable to GPCR mediated activation of the DORA-RhoA biosensor by p63RhoGEF (Fig. 1e). The recruitment of RFP-FKBP12 to the plasma membrane did not give a measurable change in YFP/CFP ratio of the RhoA biosensor, excluding effects of the rapamycin addition or membrane recruitment on the biosensor read-out.

To address the specificity of the plasma membrane as platform for RhoA activation, we interrogated other cellular endomembranes. First, as an anticipated negative control, RFP-FKBP12-cDH was recruited to a mitochondrial membrane anchor, ECFP (W66A)-FRB-MoA, (Fig. 4c), showing no increase of RhoA activity. Second, since RhoA localization is detected in the Golgi area^11^ and its activity at the Golgi is implicated in signaling^28^,^29^, we recruited RFP-FKBP12-cDH to the Golgi located anchor, FRB-ECFP (W66A)-Giantin. As can be inferred from Fig. 4d, recruiting GEF activity to the Golgi does not result in RhoA activity.

Together, these results show that recruitment of the catalytic DH domain of p63RhoGEF from the cytosol to the plasma membrane is sufficient for rapid and sustained activation of its downstream effector RhoA, highlighting a specific role for the plasma membrane as platform for RhoA activation.

### Recruitment of the DH domain of p63RhoGEF to the plasma membrane causes translocation of transcription factor MKL2 to the nucleus

Activation of RhoA leads to ROCK1/2 and mDia induced cytoskeletal re-arrangements, including actin polymerization and the formation of stress-fibers^2^ (Fig. 2), as well as the transcription of several SRF-related genes^30^. Since it is challenging to quantitatively measure the process of actin polymerization in living cells, we instead used the transcription factor Megakaryoblastic Leukemia 2 (MKL2) as a read-out. It has been shown that MKL2 can bind three G-actin molecules through its RPEL motifs, and that actin polymerization leads to dissociation of MKL2 and G-actin and subsequent translocation of MKL2 to the nucleus^30^,^31^. This transcription factor thus senses the G-actin/F-actin balance in cells and its translocation to the nucleus can be used as read-out for actin polymerization^31^,^32^.

**Figure 5:**
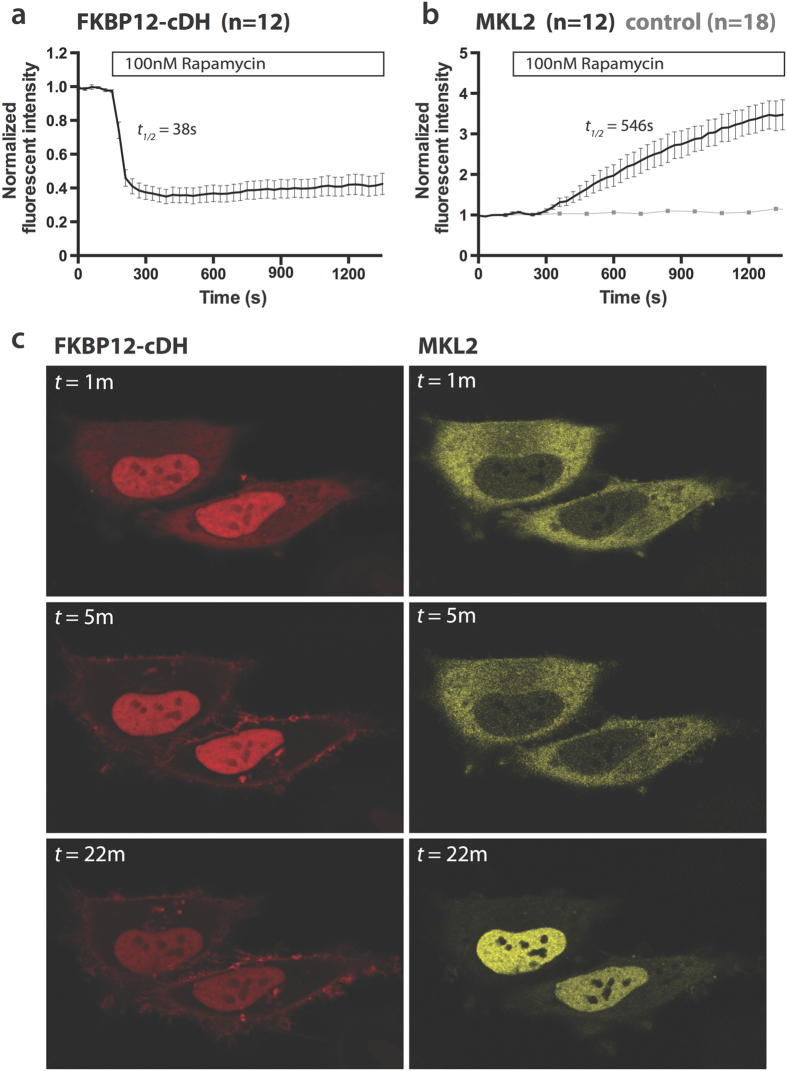
Recruitment of the DH-domain of p63RhoGEF to the plasma membrane causes translocation of transcription factor MKL2 to the nucleus. HeLa cells transfected with YFP-MKL2, Lck-FRB-CFP and RFP-FKBP12-cDH (*n* = 12) or RFP-FKBP12 (*n* = 18) were stimulated with Rapamycin (100 nM) at *t* = 150 s. (**a**) Average time-traces of rapamycin induced translocation of RFP-FKBP12-cDH to the plasma membrane (*n* = 12), as measured by loss of fluorescence in a region of interest in the cytosol. (**b**) Average time-traces of RFP-FKBP12-cDH induced YFP-MKL2 translocation to the nucleus in the same cells as (**a**), as measured by a region of interest in the nucleus. In *light grey* time-traces are shown for the same experiment performed in RFP-FKBP12 transfected control cells. (**c**) Representative images at three time intervals of RFP-FKBP12-cDH translocation to the plasma membrane and MKL2 translocation to the nucleus in single cells from the experiment shown in (**a,b**). Width of the individual images in (**c**) corresponds to 102 μm.

To investigate the effect of rapamycin-induced plasma membrane recruitment of p63RhoGEF activity on actin polymerization, we transfected HeLa cells with YFP-MKL2, Lck-FRB-CFP and either RFP-FKBP12-cDH or RFP-FKBP12. The effect of rapamycin-induced recruitment of RFP-FKBP12-cDH on the location of YFP-MKL2 was assessed with confocal microscopy. Recruitment of RFP-FKBP12-cDH to the plasma membrane resulted in nuclear translocation of MKL2 in 58% of the cells (*n* = 40). In the control condition, where only RFP-FKBP12 was relocated to the membrane, none of the cells showed MKL2 translocation to the nucleus (*n* = 30). We analyzed the kinetic parameters of this response in a subset of these cells (i.e. non-moving cells that showed both a clearly detectable rapamycin response and MKL2 response). The rapamycin induced recruitment of RFP-FKBP12-cDH was very quick with an average *t*_*½*_ = 38 ± 6 s (Fig. 5a). Nuclear translocation of YFP-MKL2 was much slower, with *t*_*½*_ = 546 ± 119 s (Fig. 5b,c, Supplemental Movie 1). The kinetics of MKL2 translocation to the nucleus are similar to previously measured GPCR mediated kinetics of this transcription factor in mammalian cells^33^. Together, these results show that recruitment of the DH domain to the plasma membrane is sufficient to induce translocation of the MKL2 transcription factor to the nucleus.

### Recruitment of the DH-domain of p63RhoGEF to the plasma membrane causes neurite retraction in N1E-115 cells, and coincides with localized RhoA biosensor activity

Recruitment of the DH domain to the plasma membrane is sufficient to induce RhoA activity and downstream signaling responses including actin polymerization and translocation of the transcription factor MKL2 to the nucleus. To test if we could use local activation of RhoA to modulate a physiological response, we turned our attention to N1E-115 cells. It has previously been shown that following LPA stimulation, these cells activate endogenous RhoA to induce F-actin modulation, neurite retraction and cell rounding^34^,^35^. However, many processes are activated upon LPA addition, making it difficult to isolate the effect of a single Rho GTPase from other signaling proteins and second messengers such as Ca^2+^. Our synthetic GEF recruitment approach allows us to study the specific effects of increased RhoA activity at the plasma membrane.

We transfected N1E-115 cells with Lck-FRB-ECFP (W66A), the DORA RhoA biosensor and either RFP-FKBP12-cDH or RFP-FKBP12. To induce N1E-115 differentiation and the outgrowth of neurites, cells were serum starved for at least 12 hours prior to the start of the experiment.

**Figure 6:**
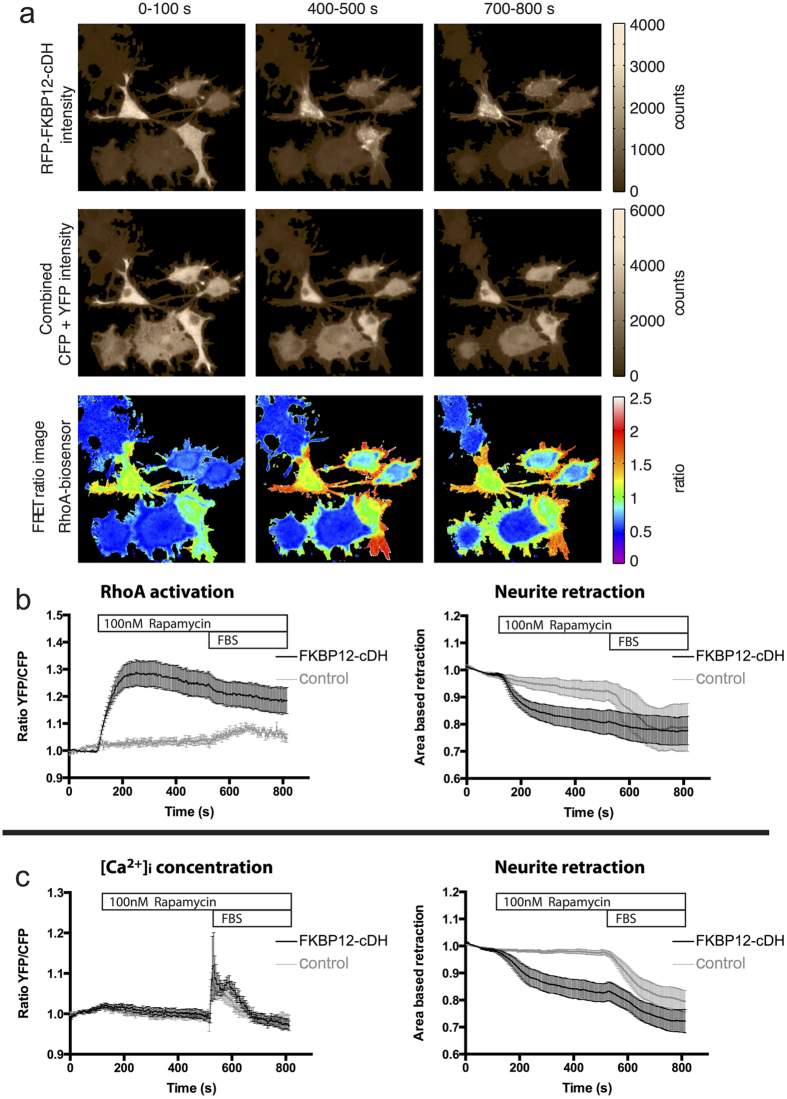
Recruitment of the DH-domain of p63RhoGEF to the plasma membrane causes neurite retraction in N1E-115 cells, and coincides with localized RhoA biosensor activity. (**a**) Example of a single N1E-115 retraction and RhoA FRET biosensor experiment. Cells were transfected with DORA-RhoA biosensor, Lck-FRB-CFP (W66A) and RFP-FKBP12-cDH and stimulated with Rapamycin (100 nM) at *t* = 125 s and Fetal Bovine Serum (FBS) at *t* = 540 s. Integrated RFP intensity images, added CFP + YFP intensity images and YFP/CFP FRET ratio images are shown of the RhoA-biosensor for three different time intervals (0–100 s, 200–300 s, 700–800 s). (**b**) Average ratiometric FRET measurements of activated DORA-RhoA biosensor and the corresponding average retraction measurements in N1E-115 cells transfected with RhoA-biosensor, Lck-FRB-CFP (W66A) and RFP-FKBP12-cDH (*n* = 8) or in RFP-FKBP12 transfected control cells (*n* = 6, *grey*). (**c**) Average ratiometric FRET measurements of intracellular Ca^2+^ and corresponding average retraction measurements in N1E-115 cells transfected with YC3.60 biosensor, Lck-FRB-CFP (W66A) and RFP-FKBP12-cDH (*n* = 8) or in RFP-FKBP12 transfected control cells (*n* = 14, *grey*). N1E-115 cells were stimulated with Rapamycin (100 nM) at *t* = 125 s and FBS at *t* = 540 s. Time traces show the average ratio change of YFP/CFP fluorescence (±s.e.m), or the average retraction of neurites. For details of the cell segmentation and retraction quantification methods, see Supplemental Fig. S4. Width of the individual images in (**a**) corresponds to 163 μm.

Following rapamycin addition, we monitored membrane recruitment of RFP-FKBP12-cDH or RFP-FKBP12, cell morphology and the fluorescence intensities of the DORA-RhoA biosensor over time, using confocal microscopy. To induce maximal neurite retraction, fetal bovine serum (FBS) was added at the end of each experiment as a positive endpoint control. The area of the individual segmented cells (see Supplemental Fig. S4 and the Methods section for a detailed description of the segmentation method) over time was used to measure overall retraction of N1E-115 neurites. In order to obtain spatial information of the RhoA biosensor, also ratio images were calculated over time. Images from a single experiment are shown in Fig. 6a, for three different time points in the experiment.

Rapamycin induced recruitment of RFP-FKBP12-cDH to the plasma membrane caused retraction of neurites (10–20% reduction in cell area) within 70–90 seconds. The retraction of neurites was accompanied by a significant increase (25–40%) in YFP/CFP ratio of the RhoA biosensor. This increase in YFP/CFP ratio was the highest in peripheral regions, indicating RhoA activity in the retracting neurite tips (Fig. 6b, Supplemental Movie 2).

Control cells expressing RFP-FKBP12 did not show retraction or an increase in RhoA biosensor activity, indicating a specific effect of the DH-domain recruitment to the plasma membrane, and excluding a possible effect of rapamycin addition (Fig. 6b). Control cells did show neurite retraction (10–20%) after subsequent stimulation with FBS (Fig. 6b).

Since stimuli that result in neurite retraction often increase Ca^2+^ levels, it is difficult to separate the effects of RhoA activity from Ca^2+^ signaling effects. To show that the effects on neurite retraction are direct and not via cross-talk with Ca^2+^ release pathways, we performed an identical experiment, but transfected the YC3.60 ratiometric Ca^2+^ concentration probe^36^ instead of the RhoA-biosensor, to assess Ca^2+^ release in the cytosol.

After stimulation with rapamycin, none of the cells transfected with either RFP-FKBP12-cDH or RFP-FKBP12 showed a change in YFP/CFP ratio of YC3.60 probe, showing a direct link between RFP-FKBP12-cDH mediated neurite retraction in N1E-115 cells, without the involvement of Ca^2+^ mediated pathways (Fig. 6c). Again as a control, FBS was added towards the end of the experiment, which resulted in a clear intracellular Ca^2+^ release in both RFP-FKBP12-cDH and RFP-FKBP12 conditions (10–20% ratio change) (Fig. 6c).

The similar fast retraction of neurites observed in the calcium imaging experiment shows that activation of endogenous RhoA is sufficient for the retraction (Fig. 6c).

Altogether, these results prove that recruiting the catalytic domain from p63RhoGEF to the plasma membrane locally increases RhoA-GTP and is sufficient for neurite retraction in N1E-115 cells. The retraction is independent of intracellular Ca^2+^ release.

## Discussion

Up to now it has been unclear when and where p63RhoGEF activates RhoA in living cells. Using the DORA-RhoA FRET based biosensor, we demonstrate the kinetics of GTP loading of RhoA by p63RhoGEF via the Gαq class of heterotrimeric G proteins at the plasma membrane in living cells. Furthermore, using several read-outs based on RhoA activity, actin polymerization and changes in cellular morphology, we demonstrate that localized GEF activity at the plasma membrane results in actin polymerization, whereas cytoplasmic GEF activity does not.

Using the DORA-RhoA biosensor, we observe very fast activation of RhoA by p63RhoGEF with a *t*_*½*_ of 21 ± 0.7 s. Interestingly, the GPCR mediated activation of a GTPase at the plasma membrane is substantially faster than the activation of a receptor tyrosine kinase mediated GTPase like HRAS at the plasma membrane, which has a *t*_*½*_ in the order of minutes^37^. The kinetics of RhoA GTP loading by p63RhoGEF is an order of magnitude faster than the kinetics reported previously *in vitro*^16^. Given that the *in vitro* measurements were performed in absence of membranes and regulatory proteins like Rho GDIs and Rho GAPs, this difference in kinetics is probably methodological, and highlights the importance of conducting these experiments in living cells^38^.

Since p63RhoGEF is located and activated at the plasma membrane^20^, our findings suggest that p63RhoGEF integrates the GPCR induced Gαq activity and uses this information to control the level of RhoA-GTP at and near the plasma membrane. The potential to rapidly activate and de-activate GEF activity upon initiation and termination of GPCR signaling, respectively, allows dynamic temporal control over RhoA activity and actin polymerization.

It has been reported *in vitro* that removal of the autoinhibitory PH domain from p63RhoGEF results in strong GEF activity, bypassing the requirement of Gαq for activation^16^,^17^. We observe this effect directly in living cells by the response on a RhoA based biosensor, showing elevated GEF activity of both pmDH and cDH. The spatial analysis of RhoA activity shows a striking difference between the membrane bound GEF, pmDH, and the cytoplasmic GEF, cDH. Since this data is recorded in widefield modus, and the contribution of biosensor in the cytoplasm in the cell cortex area is greater then the contribution of plasma membrane located biosensor in the cell body area, the measured values probably give an underestimation of the true difference between the two conditions. This membrane proximal gradient of GTP loaded RhoA exists only by virtue of spatial separation between the activating GEF activity and the inactivating GAP activity, a principle that has been described before for phosphorylated cytoplasmic proteins with rapid diffusion^39^. To fully understand the molecular details that underlie the shape of the observed gradient, it is necessary to identify the subcellular location and activity of the GAP. Another factor that determines the steepness of the gradient is the diffusion rate. In the case of RhoGTPases, the diffusion rate depends on the interaction with RhoGDIs and the interaction with membranes by virtue of their prenylated C-terminus. Both interactions in turn depend on the identity of the bound nucleotide. Due to this complexity, it is at present unclear how the mobility of RhoGTPases shapes the gradient. Since only the membrane bound GEF causes actin polymerization and stress fiber formation, we conclude that membrane located RhoA-GTP drives actin polymerization.

It is of note that the DH domain of p63RhoGEF shows specificity towards the whole RhoA family *in vitro*, including RhoB and RhoC^16^. Although we used a biosensor specifically designed to measure GTP loading on RhoA, it is not excluded that the DH domain of p63RhoGEF also activates RhoB and/or RhoC in our experiments.

In the experiments with overexpression of the pmDH construct or very high overexpression of the cytoplasmic DH construct we observed a clear effect on cell viability. The pmDH construct is essentially a constitutive active variant of p63RhoGEF, which results in a continuous activation of RhoA at the PM, causing cell rounding and cell death at too high concentrations. Moreover, we detected a pool pmDH in intracellular structures, which makes it difficult to directly compare levels of DH and pmDH expression based on fluorescent intensity. To overcome these cell viability issues and to circumvent expression level differences, we turned to the rapamycin-based dimerization system. The conditional plasma membrane localization of the DH domain conferred by rapamycin-induced heterodimerization allows straightforward comparison between its GEF activity in the cytosol and at the plasma membrane.

Previously reported recruitment strategies for GEFs often used complete Rho GEFs or at least the DH-PH tandem^40^,^41^,^42^,^43^. The GEF activity towards RhoA of the DH-domain of Gα_12_ mediated Rho GEFs (LARG, p115RhoGEF and PDZ RhoGEF) was shown to influenced by the adjacent PH domain^44^. Here, we use only the relatively small DH domain (192 amino acids) that has high constitutive GEF activity, simplifying the interpretation since we exclude effects of the PH domain or other regulatory sequences.

We propose two general mechanisms to explain the increase in actin polymerization by membrane located GEF activity, which both play a role. First, concentrating GEF activity on a 2D surface (i.e. the membrane) increases the chance of interaction with Rho GTPases. By localizing proteins on the plasma membrane or endomembranes, their local concentration is increased due to the spatial confinement in a 2D environment^45^. This local concentration effect, which increases the number of active complexes, enables the membrane environment to (temporarily) enhance signaling efficiency by two orders of magnitude^45^. However, the increase in concentration is not sufficient, since we demonstrate that localizing GEF activity at the Golgi membrane or mitochondrial membranes does not result in RhoA activity. Second, activating RhoA at the plasma membrane induces spatially restricted activation of its downstream effectors. From our studies it is not evident which of the many RhoA effectors, PKN, citron kinase, ROCK, mDia^46^ is responsible for actin polymerization and neurite retraction, this will be an interesting avenue for further research.

The subcellular location of many Rho GEFs is still poorly described and it is unclear how their location relates to activity towards RhoA. Jaiswal and colleagues recently classified 30 out of 57 Rho-, Rac and cdc42 specific Rho GEFs as mediating GTP exchange on RhoA^14^. It is very likely that some of those Rho GEFs exert their GEF function in specific subcellular compartments and under specific signaling conditions since Rho GEFs often have additional regulatory domains outside one or more canonical DH-PH cassettes^7^.

Our approach of manipulating the subcellular location of a Rho GEF and directly quantifying its activity with a FRET sensor provides a powerful strategy to reveal the spatial requirements of Rho GEF localization for nucleotide loading onto RhoA. This approach can be generally applied to different Rho GEF/Rho GTPases pairs. Here we demonstrate plasma membrane, mitochondria and Golgi membrane recruitment, but the modular architecture of the system allows recruitment to various subcellular compartments, structures or organelles.

There are some examples where the subcellular localization of specific Rho GEFs has been coupled to their function. GEF-H1 was shown to induce localized GTP loading on RhoA in the cytokinetic furrow of dividing HeLa cells^47^ and implied in HeLa cell migration through localized activation at the leading edge^48^. ECT2 is implicated in localized activation of RhoA in both the cytokinetic furrow in HeLa cells^49^ during cytokinesis and on cell-cell junctions during interphase, with a key role being proposed for the centralspindlin complex as regulator of these localized processes^50^. Our experiments in the neuronal-like N1E-115 cells demonstrate that recruiting RhoGEF activity to the periphery of these neuroblastoma cells results in localized RhoA activation at the plasma membrane, which is sufficient to induce neurite retraction.

In short, by combining a novel optimized FRET based RhoA biosensor with a synthetic biology approach to gain control over subcellular localization of GEF activity towards RhoA, we show that plasma membrane localization of RhoGEF activity is sufficient for actin polymerization. In general, our results demonstrate that the subcellular localization of GEF activity should be regarded as a crucial parameter for the regulation of Rho GTPases and subsequent cellular output.

## Methods

### Construction of fluorescent protein fusions

Mammalian expression vectors are all based on pEGFP-C1 plasmids (Clontech), in which EGFP was replaced by the YFP variant mVenus. The red fluorescent protein monomeric Cherry (mCherry) was used as RFP variant in this paper.

PCR products were ligated into mVenus-C1 plasmids by cutting the vector and PCR product with restriction enzymes BsrGI and KpnI. Restriction sites are marked in bold in primer sequences. In all cases, full length p63RhoGEF^16^ was used as a template for PCR. To construct YFP-cDH (amino acid 155–347 of p63RhoGEF), p63RhoGEF was amplified using forward primer 5′- GC**TGTACA**AGTCCAAGAAGGCTCTGGAAAGG-3′ and reverse primer 5′ - AC**GGTACC**TTAGCCCTCAAATCCCCGCAA-3′. To construct YFP-pmDH (amino acid 1-347 of p63RhoGEF), p63RhoGEF was amplified using forward primer 5′ - GC**TGTACA**AGTCCCGGGGGGGGCACAAAGGG-3′ and reverse primer 5′-AC**GGTACC**TTAGCCCTCAAATCCCCGCAA-3′.

RFP variants of these constructs were made by color swapping the mVenus with mCherry with restriction enzymes AgeI and BsrGI.

The RFP-p63RhoGEF and RFP-p63RhoGEF^1^,^2^,^3^,^4^,^5^,^6^,^7^,^8^,^9^,^10^,^11^,^12^,^13^,^14^,^15^,^16^,^17^,^18^,^19^,^20^,^21^,^22^,^23^,^24^,^25^,^26^,^27^,^28^,^29^ (amino acid 1–29 of p63RhoGEF) were obtained by cutting the mVenus variants described earlier^20^ with AgeI and BsrGI and exchanging mVenus for mCherry.

RFP-FKBP12-C1 was obtained as previously described^20^.

The RFP-FKBP12-cDH was obtained by cutting RFP-FKBP12-C1 with MfeI and Acc651 and inserting the DH domain cut from the RFP-cDH vector with MfeI and BsrGI. A schematic overview of the constructs is depicted in Supplemental Fig. S1.

A Dimerization Optimized Reporter for Activation (DORA) single-chain RhoA biosensor was constructed such that GTP-loading of RhoA is translated into fluorescent protein heterodimerization, thereby increasing FRET.

The DORA-RhoA coding sequence within a pTriEx backbone is MAHHHHHHGSGS-cpPKN-GTGS-cpV-L9H-L9H-L9H-GS-Cer3(1–229)-AS-RhoA. The lay-out is analogous to a previously published RhoA probe^21^, retaining regulation by Rho GDIs. Introducing the Q63L mutation in RhoA, locking RhoA in the GTP-bound state and mutating PKN1 (L59Q), preventing binding of RhoA, respectively, resulted in constitutive active (RhoA_sensor_-ca) and non-binding (RhoA_sensor_-nb) sensors. The detailed development of the sensor will be described elsewhere.

pTriExRhoA1G and pTriExRhoA2G (Addgene plasmid # 40176) were a gift from Olivier Pertz.

EGFP-MKL2 was a kindly provided by J.S. Hinson^33^. We swapped the EGFP for mVenus with restriction enzymes AgeI and BsrGI.

The Lck-FRB-ECFP (W66A) and Lck-FRB-ECFP were a kind gift from M. Putyrski^51^, FRB-YFP-Giantin and CFP-FRB-MoA were a kind gift from T. Inoue^52^. We swapped the YFP in FRB-YFP-Giantin for ECFP (W66A) with restriction enzymes AgeI and BsrGI. We swapped the CFP in CFP-FRB-MoA for ECFP (W66A) with restriction enzymes NheI and BsrGI. A bacterial expression plasmid RSET-YCam 3.6 was a kind gift of A.Miyawaki^36^. To enable expression in eukaryotic cells we cut the YCam 3.6 coding sequence from the plasmid with NheI and EcoRI and inserted the fragment in a Clontech-style pEGFP-C1 plasmid, cut with the same enzymes.

### Cell Culture & Sample Preparation

HeLa cells (American Tissue Culture Collection: Manassas, VA, USA) and N1E-115 Neuroblastoma cells (European Collection of Cell Cultures: Salisbury, UK) were cultured using Dulbecco’s Modified Eagle Medium (DMEM) supplied with Glutamax, 10% FBS, Penicillin (100 U/ml) and Streptomycin (100 μg/ml). All cell culture media were obtained from Invitrogen (Bleiswijk, NL).

Cells were transfected in a 35 mm dish holding a glass 24 mm Ø #1 coverslip (Menzel-Gläser, Braunschweig, Germany), using 1–2 μl Lipofectamine 2000 according to the manufacturer’s protocol (Invitrogen), 0.5–1 μg plasmid DNA and 50 μl OptiMeM (Life Technologies, Bleiswijk, NL). N1E-115 cells were transfected in OptiMeM to accomplish neurite outgrowth by serum starvation^53^.

Samples were imaged 1 day after transfection unless stated otherwise. After overnight incubation at 37 °C and 5% CO_2_, coverslips were mounted in an Attofluor cell chamber (Invitrogen, Breda, NL) and submerged in microscopy medium (20 mM HEPES (PH = 7.4), 137 mM NaCL, 5.4 mM KCL, 1.8 mM CaCL_2_, 0.8 mM MgCl_2_ and 20 mM glucose). All live cell microscopy was done at 37 **°**C.

### Widefield microscopy

Ratiometric FRET measurements in HeLa cells were performed using a wide-field fluorescence microscope (Axiovert 200 M; Carl Zeiss GmbH) kept at 37 °C, equipped with an oil-immersion objective (Plan-Neo- fluor 40×/1.30; Carl Zeiss GmbH) and a xenon arc lamp with monochromator (Cairn Research, Faversham, Kent, UK). Images were recorded with a cooled charged-coupled device camera (Coolsnap HQ, Roper Scientific, Tucson, AZ, USA). Typical exposure times ranged from 50 ms to 400 ms, and camera binning was set to 4 × 4. Fluorophores were excited with 420 nm light (slit width 30 nm) and reflected onto the sample by a 455DCLP dichroic mirror and CFP emission was detected with a BP470/30 filter, and YFP emission was detected with a BP535/30 filter by rotating the filter wheel. In the static experiments, YFP was excited with 500 nm light (slit width 30 nm) and reflected onto the sample by a 515DCXR dichroic mirror and emission was detected with a BP535/30 filter. RFP was excited with 570 nm light (slit width 10) and reflected onto the sample by a 585CXR dichroic mirror and emission of RFP was detected with a BP620/60 filter. All acquisitions were corrected for background signal and bleedthrough of CFP emission in the YFP channel (around 55% of the intensity measured in the CFP channel).

In the static experiments described in Fig. 3 the experimental data was binned based on RFP fluorescence intensity (50–300 a.u. after background substraction), in order to compare similar concentrations between conditions. In dynamic experiments, cells were stimulated with 100 μM Histamine (Sigma-Aldrich) and 10 μM Mepyramine (Sigma-Aldrich) or 100 nM Rapamycin (LC Laboratories, Woburn, USA). The specific Gαq inhibitor UBO-QIC (FR900359) was added to the cells 2 hours before the measurements started at a concentration of 2 μM and was purchased from the University of Bonn (http://www.pharmbio.uni-bonn.de/signaltransduktion).

### Confocal microscopy

HeLa cell and N1E-115 rapamycin recruitment and ratiometric FRET experiments were performed using a Nikon A1 confocal microscope equipped with a 60x oil immersion objective (Plan Apochromat VC, NA 1.4). The pinhole size was set to 5 Airy units to obtain semi-widefield optical slices (±3.5 μm) in the ratiometric FRET and N1E-115 experiments, and set to 1 Airy unit in the MKL2 translocation experiments (<0.8 μm).

For the ratiometric FRET experiments, samples were sequentially excited with 447 nm and a 561 nm laser line, and reflected onto the sample by a 457/514/561 dichroic mirror. CFP emission was filtered through a BP482/35 emission filter; sensitized YFP emission was filtered through a BP525/50 emission filter; RFP emission was filtered through a BP595/50 emission filter. All acquisitions were corrected background signal and for bleedthrough of CFP emission in the YFP channel (around 32% of the intensity measured in the CFP channel).

For the recruitment experiments: 447 nm, 514 nm and 561 nm laser lines were reflected onto the sample by a 457/514/561 dichroic mirror. To avoid bleed-through, images were acquired with sequential line scanning modus.

CFP, YFP and RFP emission was detected using BP482/35, BP540/30 and BP595/50 emission filters respectively. In dynamic experiments, cells were stimulated with 100 nM Rapamycin (LC Laboratories, Woburn, USA) and 10% FBS (final concentration) at the indicated time points. Images are representative of multiple experiments, performed on different days.

### Spectral imaging

One day after transfection, cells were excited with light from a mercury Arc lamp passed through a 436/10 nm excitation filter. The emission was passed through a LP460 long pass filter and an imaging spectrograph (Imspector V7, Specim, Finland). Spectral images were acquired with a CCD camera (ORCA ER, Hamamatsu, Japan). Subsequently, an image was acquired from the same field of view to quantify relative YFP intensity (excitation at 500/20 nm and emission passed through a 534/20 nm filter). Each full emission spectrum was divided by the averaged YFP emission intensity, thereby correcting for differences in expression levels. The single cell spectra were used to calculate the average emission spectrum.

### Fluorescence lifetime imaging microscopy

Frequency domain FLIM was performed at a frequency of 75.1 MHz and was done as described before^54^.

### Actin staining

HeLa cells transfected with different p63RhoGEF constructs were washed with phosphate-buffered saline solution (PBS) and fixed with 4% formaldehyde for 20 minutes. After washing with PBS, cells were permeabilized with PBS containing 0.2% Triton X-100. After a second wash step with PBS and blocking of non-specific binding by 1% BSA in PBS for 10 minutes, cells were stained with 0.1 μM TRITC-phalloidin (Sigma-Aldrich) and 0.1 μg/ml DAPI. After washing with PBS, cells were mounted in Mowiol and fluorescence images were obtained using a widefield fluorescence microscope (Axiovert 200 M; Carl Zeiss GmbH).

### Data Analysis

ImageJ (National Institute of Health) was used to analyze the raw microscopy images. Further processing of the data was done in Excel (Microsoft Office) and graphs and statistics were conducted using Graphpad version 6.0 for Mac, GraphPad Software, La Jolla California USA, www.graphpad.com.

The *t*_*½*_ is defined as the time between agonist addition and the time at which the response reached 50% of the maximum value. Boxplots in Fig. 1, Fig. 3 and Supplemental Fig. S2 were generated online, using the website http://boxplot.tyerslab.com/. The FRET and/or retraction data in Figs 3 and 6 were analyzed using a MatLab script (MATLAB, The MathWorks, Inc., Natick, Massachusetts, United States). Prior to any of the ratiometric FRET analyses donor CFP and acceptor YFP channels were background corrected by subtracting the modal pixel value and both channels were aligned to each other. Details of data analysis performed in Fig. 3 can be found in Supplemental Fig. S3. Prior to watershed segmentation of HeLa cells, cells were manually selected by adding seed points and touching cells were separated by manually drawing boundaries between them. A local threshold was applied to the watershed region such that it only included pixels that were higher than 15% of the maximum intensity in that region. For all channels the mean fluorescence intensity was calculated for each segmented region. Ratiometric FRET analysis was applied to each segmented region in the cell. For Fig. 6, detailed methods are supplied in Supplemental Fig. S4. Because N1E-115 cells do not migrate over the time course of the experiment, starting regions, each containing a single cell, were obtained by watershed segmentation of the summed time series. For every time point a threshold (fivefold background s.d.) was applied to each starting region in order to obtain a cell region. In order to capture retraction of the cells robustly we subsequently applied another local threshold and obtained the cell area per time point, within the cell region that was higher than 10% of the 99-percentile fluorescence intensity. For each thresholded cell region and time point, FRET ratios were calculated based on the median intensity values. The recruitment response induced by rapamycin observed in the RFP-BP channel was based on the median fluorescence value in the center of the cell regions; the center was obtained by eroding the cell region by 9 pixels. MatLab scripts are available on request.

## Additional Information

**How to cite this article**: Unen, J. *et al.* Plasma membrane restricted RhoGEF activity is sufficient for RhoA-mediated actin polymerization. *Sci. Rep.* **5**, 14693; doi: 10.1038/srep14693 (2015).

## Supplementary Material

Supplementary Information

Supplementary Movie 1

Supplementary Movie 2
