# Supplementary material for: Plasma membrane restricted RhoGEF activity is sufficient for RhoA-mediated actin polymerization
Source: Sci Rep. 2015 Oct 5;5:14693. doi: 10.1038/srep14693 (PMC4592971; doi:10.1038/srep14693)
Supplement: Supplementary Information [file srep14693-s1.pdf]

Plasma membrane restricted RhoGEF activity is sufficient for RhoA-mediated actin polymerization

Jakobus van Unen[*], Nathalie R. Reinhard[*], Taofei Yin[†], Yi I. Wu[†], Marten Postma[*], Theodorus W.J. Gadella Jr.[*], Joachim Goedhart[*, ‡]

Supplemental Figure S1

Schematic overview of the p63RhoGEF constructs used in this study

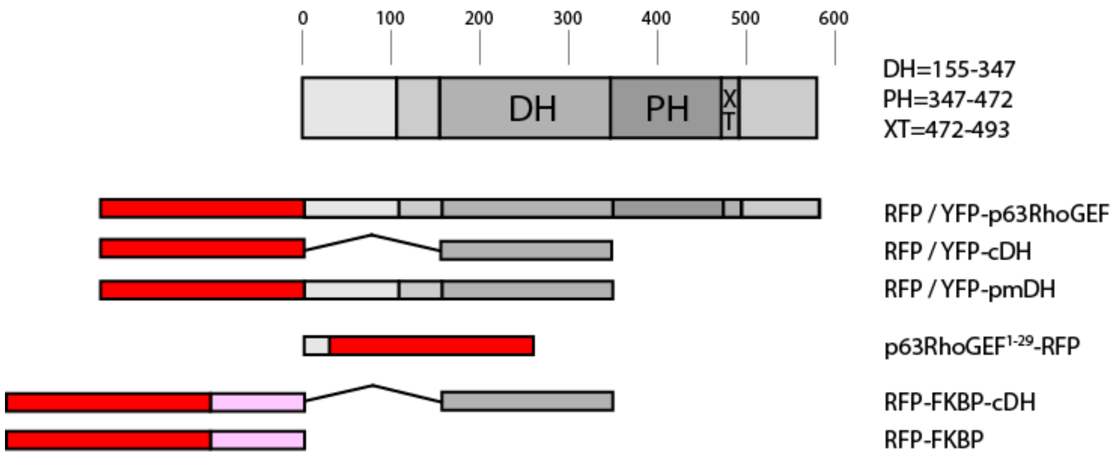

Supplemental Figure S1:

Full-length p63RhoGEF comprises of 580 amino acids. The Dbl homology (DH) and pleckstrin homology (PH) domain, identified based on their homology with other proteins, are indicated. Also the C-terminal extension of the PH domain is indicated (XT). Synthetic constructs comprising only subsets of the p63RhoGEF domains were made to assess their function: cDH (a.a. 155-347), pmDH (a.a. 1-347) and p63RhoGEF$^{1-29}$ (a.a. 1-29). The red fluorescent protein variant that was used is mCherry, drawn to scale (236 amino acids) and indicated by red rectangles. The yellow fluorescent protein variant that was used is mVenus (238 amino acids). For the rapamycin recruitment assays, mCherry-FKBP12-cDH and mCherry-FKBP12 (drawn to scale) were constructed, which can form a ternary complex with FRB and rapamycin.

Supplemental Figure S2

Expression of the cDH and pmDH domain of p63RhoGEF leads to high GEF activity in HEK293 cells

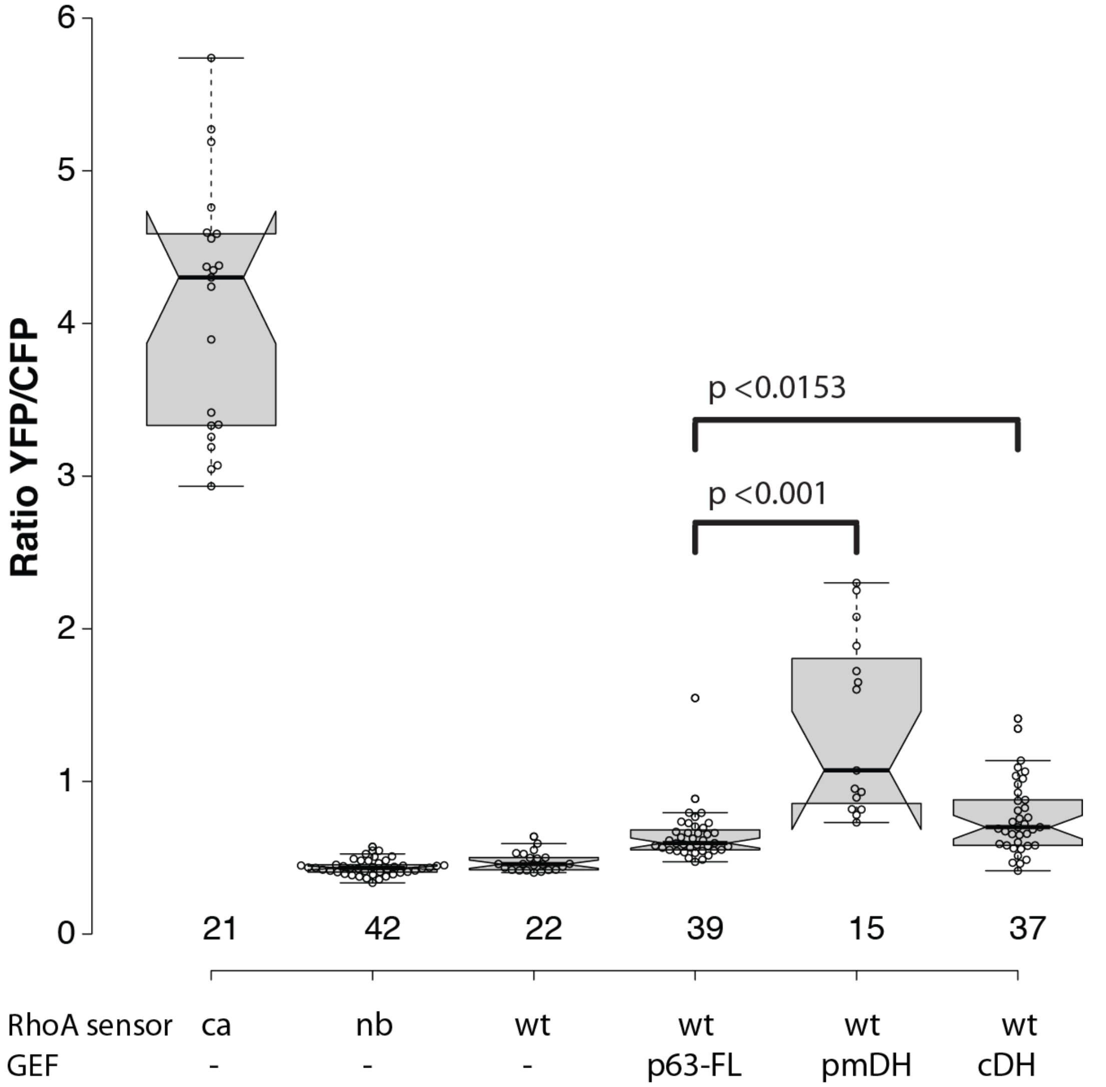

Supplemental Figure S2:
Boxplot showing YFP/CFP ratio of the DORA-RhoA biosensor in HEK293 cells. Cells transfected with the constitutive active (ca, *n* = 21) or non-binding (nb, *n* = 42) RhoA biosensor were co-transfected with an empty vector containing just RFP to keep expression levels equal between the different experimental conditions. Wild-type (wt) RhoA-biosensor was transfected with p63RhoGEF$^{1\text{-}29}$-RFP (control, *n* = 22), RFP-p63RhoGEF (*n* = 39), RFP-pmDH (*n* = 15) and RFP-cDH (*n* = 37).
Center lines show the medians; box limits indicate the 25th and 75th percentiles as determined by R software; whiskers extend 1.5 times the interquartile range from the 25th and 75th percentiles; data points are plotted as open circles. Statistical significance between conditions was determined by performing a two-tailed Mann-Whitney test. P-values are shown in plot for the conditions with significant different medians.

Supplemental Figure S3

Description and visualization of the workflow used to produce Figure 3C

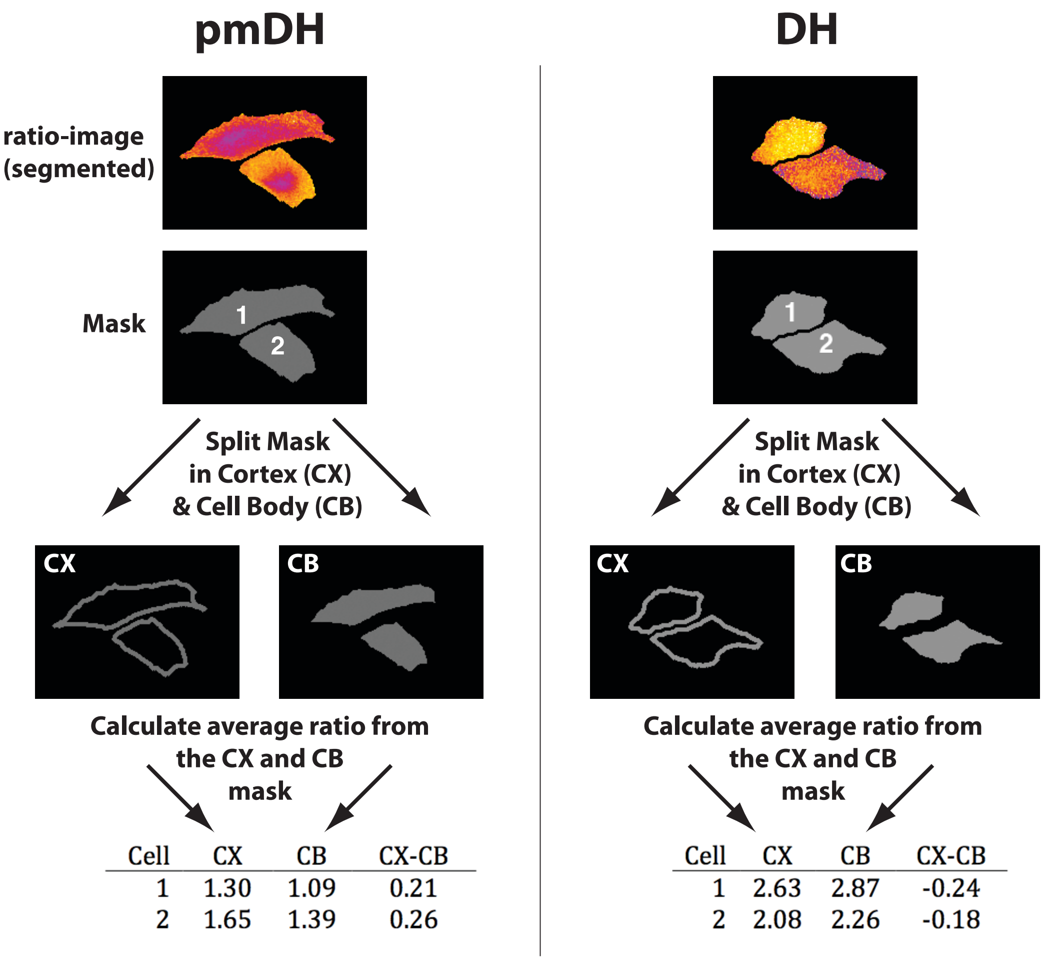

Supplemental Figure S3:
Ratio images were used to create a binary mask per cell. The mask was split into two. The outer boundary of 5 pixels wide corresponding to the cell cortex (CX) and the remaining inner part corresponding to the cell body (CB). These two masks were used to calculate the average FRET ratio corresponding to these regions. The difference between the ratio from the CX and CB is calculated as a measure of spatial inhomogeneity. A value of zero indicates no spatial differences, while a positive value indicates increased RhoA-GTP in the cortex relative to the body and a negative value denotes decreased RhoA-GTP in the cortex relative to the body.

Supplemental Figure S4

Example of the quantification of a single N1E-115 retraction experiment

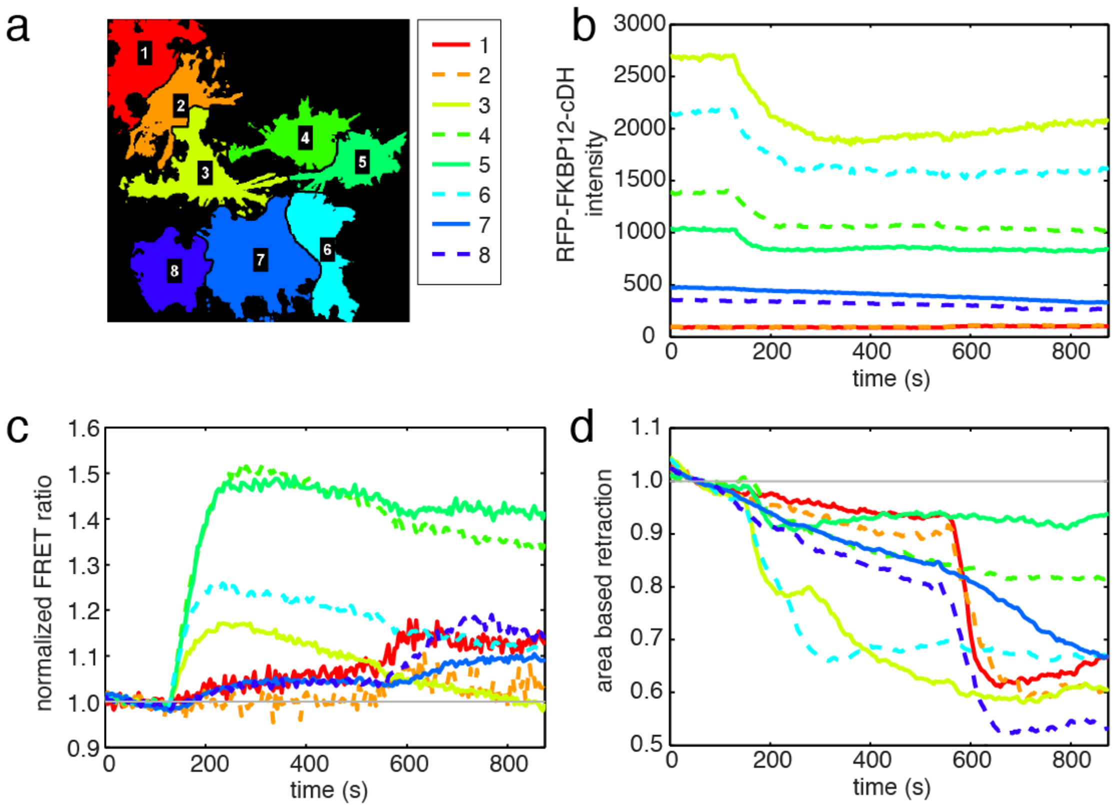

Supplemental figure S4:

Cells were prepared for the experiment as described in the main text. In order to quantify the effects of the recruitment of the DH-domain to the plasma membrane, cells were segmented by using guided watershed segmentation on an image obtained by combining all time points and channels (A). Subsequent analysis was done on the initial segmented cell regions, where the translocation of RFP-FKBP12-cDH or RFP-FKPB12 on rapamycin addition was assessed per cell with a region of interest in the cytosol (B). The RhoA FRET biosensor read-out is calculated from the YFP/CFP ratio whole cell regions (C). Neurite retraction is calculated from the whole cell region areas (D).

Supplemental Movie M1:
MKL2 translocation to the nucleus.

Relocation of the DH-domain of p63RhoGEF to the plasma membrane causes translocation of transcription factor MKL2 to the nucleus. HeLa cells transfected with YFP-MKL2, Lck-FRB-CFP and RFP-FKBP12-cDH were stimulated with Rapamycin (100nM) at t = 150 s. Time-lapse confocal imaging of the fluorescence intensity in the RFP, YFP and CFP channels. An overlay of the YFP and CFP channel is shown on the bottom-left. Width of the individual channels is 118μm, total acquisition time was 45 minutes.

Supplemental Movie M2:
N1E-115 neurite retraction and localized RhoA biosensor activation.

Relocation of the DH-domain of p63RhoGEF to the plasma membrane causes neurite retraction in N1E-115 cells, and coincides with localized RhoA-biosensor activity. Before rapamycin stimulation the DH-domain is localized in the cytoplasm and after at plasma membrane. This coincides with increased FRET activity at plasma membrane of the RhoA biosensor and subsequent retraction of neurites. Cells not expressing RFP-FKBP12-DH lack the first retraction response but show clear activity at serum stimulation (two cells in top-left corner). N1E-115 cells transfected with DORA-RhoA biosensor, Lck-FRB-CFP and RFP-FKBP12-cDH were stimulated with Rapamycin (100nM) at t =125 s and Fetal Bovine Serum (FBS) at t =540 s. Time lapse confocal imaging of the RFP channel (A), sum of CFP and YFP channels (B), thresholded YFP/CFP FRET image of RhoA-biosensor (C), intensity overlay of FRET ratio image (D). Color bars in the top-right corner per channel depict fluorescent intensity (A, B) or YFP/CFP ratio (C). Image width of the individual channels is 163 μm, total acquisition time was 14 mins and 34 s in real-time. For visualization purposes the time series were filtered with a 3D median filter with a radius of 2 pixels and only the cell regions obtained from the cell segmentation process are shown.

Supplementary Note

Comparison of RhoA sensor performance

Several parameters determine the performance of a FRET based biosensor, of which brightness, sensitivity and dynamic range (contrast) are most important. We compared existing FRET based sensors for RhoA that are based on N-terminal tagged RhoA to preserve regulation by RhoGDI and native localization via prenylation of the CaaX box at the C-terminus of RhoA. The sensors we compare include RhoA1G [1], RhoA2G [2] and DORA-RhoA (this work).

Brightness

Since RhoA2G and DORA-RhoA have the same acceptor (Venus) it is possible to compare their brightness by normalizing the donor emission and sensitized emission with directly excited Venus emission. This analysis allows direct comparison of relative emission independent of the expression level. A brightness analysis was performed on our wide-field setup by acquiring CFP emission (excitation 420/30 nm, emission 470/30 nm), sensitized emission (excitation 420/30 nm, emission 535/30 nm) and YFP emission (excitation 500/30 nm, emission 535/30 nm). The CFP and sensitized emission levels were quantified from individual cells and normalized by dividing by the YFP emission. The results are presented in panel A, showing that the normalized brightness of the TFP donor of RhoA2G is much lower than emission from the Cerulean3 donor of DORA-RhoA.

We realized that the 470/30 nm emission filter is suboptimal for detecting TFP (emission maximum 492 nm). To perform unbiased quantification of sensor brightness, independent of emission filters, we employed spectral imaging [3]. Single cell emission spectra were recorded by excitation at 436/10 nm and normalized by fluorescence of Venus (directly excited at 500 nm and detected at 534/20 nm). The resulting average emission spectra show increased (integrated) brightness (1.5-fold) of the DORA-RhoA sensor relative to the RhoA2G sensor (panel B). From these data it can be concluded that even for optimal TFP emission filter settings (480/40, [2]), the difference in brightness of the DORA-RhoA in the donor channel is more than 2-fold higher than RhoA2G.

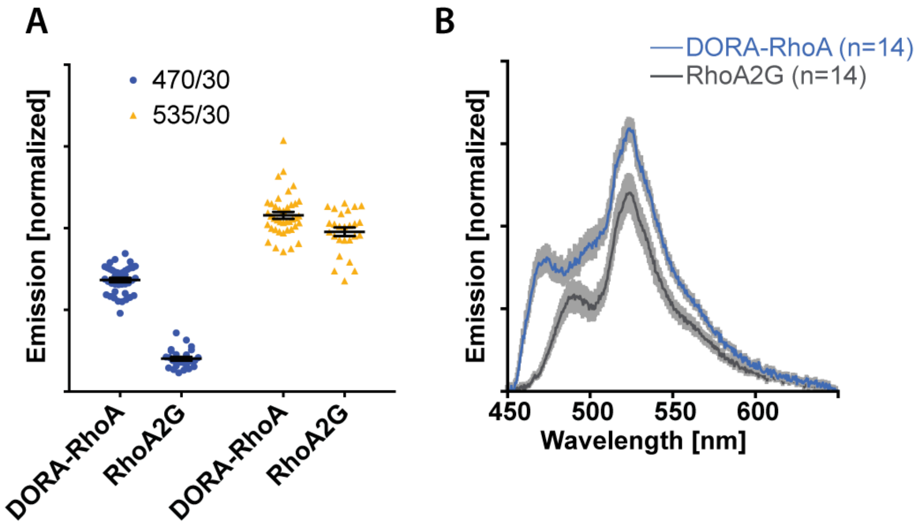

The single cell spectra were acquired with 436 nm excitation light, which is optimal for CFP variants. In contrast, for TFP 460 nm excitation light would be optimal, with a 25% increase in excitation efficiency compared to 436 nm excitation. However, a longer excitation wavelength would come at the cost of a narrower emission bandpass filter again resulting in less signal. Together, our results demonstrate that DORA-RhoA is substantially brighter than RhoA2G.

Dynamic Range

To analyze the dynamic range and sensitivity in relevant experiments we performed dynamic single cell measurements under identical conditions for RhoA1G, RhoA2G and DORA-RhoA. HeLa cells were transfected with 750 ng of sensor (each sensor is in the same TriEx-based vector) and 750 ng of p63-mCherry. The next day the cells were activated by adding 100 μM histamine and deactivated by adding 10 μM pyrilamine (similar to figure 1E in the main text). FRET ratio-imaging (identical excitation/emission settings, time intervals and exposure times) was performed to study the response of the RhoA sensor to the stimulation.

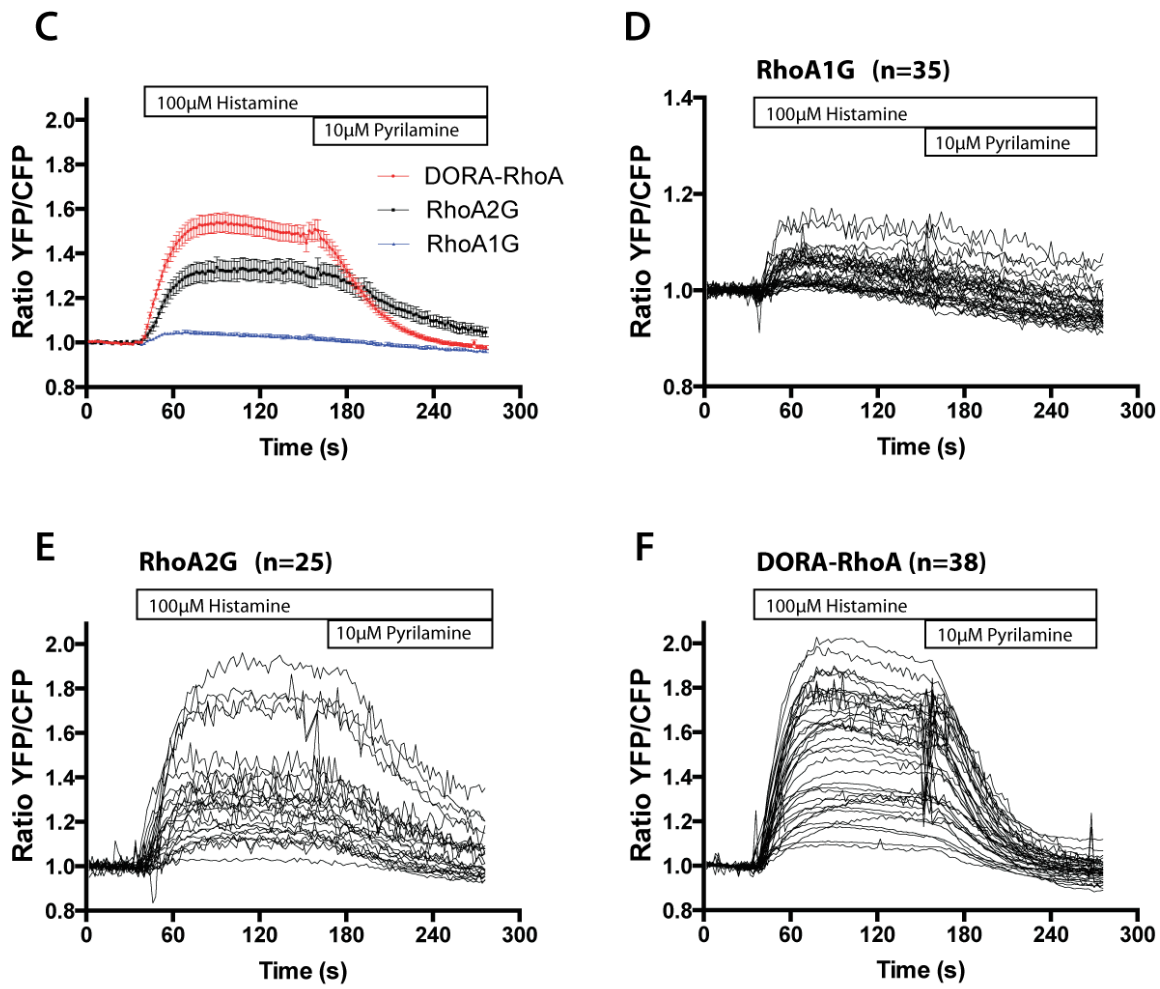

Average FRET-ratios from each sensor (error bars indicate s.e.m.) are depicted in panel C, showing hardly any response for RhoA1G, while RhoA2G and DORA-RhoA show a clear activation by histamine and a deactivation by pyrilamine. Responses acquired from individual cells are shown in panels D, E and F for

RhoA1G, RhoA2G and DORA-RhoA respectively. Strikingly, cells with RhoA1G that react significantly to histamine, do not show a clear reduction of the FRET ratio after receptor deactivation by pyrilamine. Of note, the off-kinetics of RhoA2G are somewhat slower than the off-kinetics of DORA-RhoA. In conclusion, the average dynamic range of DORA-RhoA is better than RhoA2G under relevant live cell imaging conditions.

Conclusion

In summary, judging from brightness and dynamic range, DORA-RhoA is the preferred FRET based sensor for measuring RhoA nucleotide loading state in single living cells.

<u>References</u>

1. Pertz O, Hodgson L, Klemke RL, Hahn KM: **Spatiotemporal dynamics of RhoA activity in migrating cells.** *Nature* 2006, **440**:1069–1072.

2. Fritz RD, Letzelter M, Reimann A, Martin K, Fusco L, Ritsma L, Ponsioen B, Fluri E, Schulte-Merker S, van Rheenen J, Pertz O: **A Versatile Toolkit to Produce Sensitive FRET Biosensors to Visualize Signaling in Time and Space.** *Sci Signal* 2013, **6**:rs12.

3. Goedhart J, van Weeren L, Hink MA, Vischer NOE, Jalink K, Gadella TWJ: **Bright cyan fluorescent protein variants identified by fluorescence lifetime screening.** *Nat Methods* 2010, **7**:137–139.
